# The Hospital Nursing Supplement

**Published:** 1893-05-06

**Authors:** 


					The HOSpltal% May 6, 1893. Extra. Supplement.
l^ospttal" Utttrstttg Mivvov<
Being ?hb Extra Nursing Supplement o? "Thh Hospital" Newspaper.
[Contributions (or this Supplement should be addressed to the Editor, Thh Hospital, 428, Strand, London, W.O., and should have the word
" Nursing" plainly written in left-hand top corner of the envelope.]
IRews from the IRurslng MorI5.
A ROYAL VISIT.
Their Royal Highnesses the Prince and Princess
of Wales will open the new wing of the Hospital for
Sick Children in Great Ormond Street on Jnne 24th.
The Princess has intimated her willingness ,to receive
pnrses on the occasion, and the contents of these will
be applied towards paying off the building fund debt
of ?12,000. H.R.H. the Duke of Fife is President of
the Hospital for Sick Children.
PROVISION FOR OLD AGE.
Arrangements have been made by the Committee
of the Royal Free Hospital for federation with the
Royal National Pension Fund for Nurses. It has been
resolved to pay half the premiums required out of the
hospital funds, the other part being contributed by
the nurses themselves in order to secure their pensions.
This practical method of encouraging workers to make
provision for the days of old age or sickness has already
been successfully carried out in other hospitals and
institutions.
A WELCOME GIFT.
The new Day Nursery opened by the Duchess of
Westminster on April 29th, at Plaistow, is the gift of
an anonymous friend. It is a large new building, well
adapted for the purpose, and will be doubtless of im-
mense value to the poor little folks it is designed to
shelter. Every one acquainted with the universal
poverty of this huge district can realise the small chance
which the babies have there of receiving proper care and
nourishment. The original Day Nursery, which waa
opened by Miss Katherine Twining and a friend, had
to be given up last autumn on account of its inade-
quate size.
BUILDING OPERATIONS.
The work of reconstruction has been embarked upon,
and the familiar old front of the Royal Free Hospital
in Gray's Inn Road will soon belong to the things of
the past. The bad condition of the Casualty and other
departments in the old buildings have made the altera-
tions imperatively necessary. A laundry is to be
added, and other needed improvements will doubtless
be made. The statement on this subject in the annual
report does not appear to include plans for improving
the accommodation for the nurses. Surely something is
to be done in this direction for the comfort of the re-
sident staff of nurses, and also for the private workers,
who are highly commended in the paragraph headed,
" Trained Nurses' Institute." The bath-room and the
sleeping accommodation can neither of them be con-
sidered to fulfil the modern requirements of a training
school, and the sooner this is realised by the committee
the better.
THE NURSE DOLLS.
The Nurse Dolls have accepted an invitation to the
exhibition which is to be held at the Mansion, "Victoria
Park, Keighley, next June, and they are asked to
Blackpool in September. As tlie proceeds of the
former are to go to the Cottage Hospital, it is specially
appropriate for the dolls to give their services on the
occasion. The Blackpool and Keighley Committees
have arranged to re-dresB and renovate them. How
many of the correspondents who made such kind offers
lately to help with the dolls will assist us instead to
add to The Hospital Collection of nursing uniforms ?
We want a sample of a pretty and suitable Cottage
Hospital outfit, and we shall be glad to hear from any
readers who are willing to send dolls dressed in the
following uniforms, as we wish to have the collection
made as complete as possible: Aylesbury and Barn-
staple General Infirmaries, North Lonsdale Hospital,
R. U. Hospital, Bath, Birkenhead Borough and Private
Nursing, Wirrall Children's Hospital, Birmingham
Workhouse Infirmary, Blackburn and East Lancashire
Infirmary, Bolton Infirmary, Bournemouth Royal
Yictoria Hospital, Bradford Children's Hospital, Adden-
brooke's Hospital, Chatham Hospital (Sfc. Bartholo-
mew's), Cheltenham General Hospital, Cold Ash
Children's Hospital, Coventry Hospital, Derby Royal
Infirmary, Dorset County Hospital, Noble's Isle of
Man Hospital, Dover and Durham Hospitals.
UNATTAINABLE TRAINING.
Any woman who makes up her mind to become a
nurse has a certain amount of choice in the matter of
training schools. She can select those she likes best,
with a reasonable hope that at one or other she will
eventually gain admittance, after the necessary pre-
liminaries have been gone through. When a young
man, who probably also has a certain aptitude for
the nursing profession, and a very earnest wish to
get thoroughly trained, begins to make inquiries,
he meets with nothing but discouragement. He
has thought, in a vague sort of fashion, that he
could go into a hospital and learn his work, after
the same method as a woman, but he is soon unde-
ceived. Thorough medical and surgical training is not
attainable for any would-be male nurse. No general
hospital in England, that we know of, makes any pro-
vision for instructing men. The good, all-round train-
ing which would make an intelligent, well-conducted
young man into a valuable attendant for invalids of
his own sex, is quite unattainable at present. Cer-
tainly it is only desired by a small number of
persons, and still fewer are suited to take up the
work, but it seems a pity that no institution is found
willing to take pity on them. There is little doubt
that the competent male nurse would be appreciated,
but the difficulty of his training continues insuperable.
QUEEN'S NURSES AT PORTSMOUTH.
The meeting in connection with the Portsmouth
Association for Nursing the Sick Poor, at which the
Hi 7HE HOSPITAL NURSING SUPPLEMENT. May 6,1893.
Duke and Duchess of Connaught were present a short
time ago, has had excellent results. The staff of two
nurses has been increased to four, including a Super-
intendent, and affiliation with the Q.Y.J.I, has been
also accomplished. The latter step not only secures a
supply of duly-qualified nurses, but also provides for
their systematic inspection and supervision. The
military authorities have arranged to contribute ?70
per annum to the Association, that one nurse may be
Bet apart for soldiers' families; and the naval
authorities are hoping to raise funds to secure similar
advantages for the wives and children of seamen and
marines. The good feeling and interest exhibited at
Portsmouth promise permanent success to this branch
of the Queen Victoria Jubilee Institute.
WELL-EARNED HONOURS.
A most gratifying presentation was recently made
to Mrs. S. Chambers on the occasion of her departure
from Wolverhampton Borough Hospital, where she has
held the position of Matron. The Corporation, Cor-
poration officials, and the public subscribed towards a
handsome bronze and marble clock and a purse of
gold, which was accompanied by an appropriate
address. Some of the inhabitants of Blakenhall gave
a pair of vases and a timepiece, and the nurses and
staff presented a beautiful Japanese work-table. There
were also other gifts, and each was accompanied by
earnest good wishes for Mrs. Chambers' future career
as Matron of the Guildford Infectious Hospital. Mr.
Alderman Major, J.P., Chairman of the Sanitary Com-
mittee, spoke in the highest terms of her unremitting
care and kindness to her patients and her admirable
management. Especial reference was made to the
great tact with which Mrs. Chambers had helped to
promote the good feeling now established between the
Sanitary Committee and the public. Both officially
and unofficially all classes of persons appear to have
united in bearing testimony to the good work which
Mrs. Chambers had done in their midst, and her de-
parture is universally regretted. We cordially join in
the good wisheE which follow her to her new sphere of
usefulness.
SELF-HELP.
The workmen of Stockton and Thornaby are sup-
porting their District Nursing Association most
heartily, more than half the total annual receipts
being given by themselves. It is estimated that some
7,000 men are regular subscribers to the fund. The
management and hearty co-operation of all classes are
alike admirable, and now the Convalescent Home,
Sadberge Hall, whioh has been secured for six months,
is another sign of progress. It is hoped to give about
100 women and children the benefit of a visit to the
Home during the summer. There certainly seems every
prospect of the workpeople eventually enabling their
committee to establish a permanent convalescent home
for these districts.
OLD AND INCOMPETENT.
The proposed changes in the arrangements for
nursing the sick at Cheltenham Workhouse have led
to some interesting disclosures as to the kind of
individuals who have hitherto been allowed to act as
assistant nurses. Strangely enough, until quite recently
the Visiting Committee and the Local Government
Board Inspector are said to liave reported the work-
house and infirmaries as being " properly conducted."
This statement cannot possibly have included the
subject of efficient nursing for the paupers. Doubtless
the two paid nurses, one on the male and the other on
the female side, have done what they could for all the
patients during their hours on duty, but in their
absence and at night the poor sufferers cannot have
had much comfort. The assistant nurses on the female
side, that is to say, the paupers regularly employed to
attend on the sick, consisted of one old lady of seventy-
five, suffering from chronic rheumatism, and herself
"half a patient"; another was gouty, and all were
more or less incapable and infirm. In the men's ward
the ages were still greater. The youngest, aged twenty-
six, was an imbecile, and the ages of the others ranged
from sixty-three to eighty-two. The man aged sixty-
three was " blind in one eye and could hardly see with
the other." Such a list is full of pathos, both attend-
ants and patients claiming almost equally our pity. In
the face of such facts, there was still a division of
opinion as to whether any change in existing arrange-
ments was really necessary. Although fourteen voteB
were taken in favour of the proposed reforms, nine
gentlemen were against them, and three did not vote !
TRAINING IN FEVER NURSING.
The Board of Management of the County and City
of Cork Women and Children's Hospital issued a satis-
factory report at their annual meeting, and they called
attention to a detail of their system of training nurseB,
which is peculiarly good, and might certainly be
followed with advantage by other institutions. They
have arranged with the authorities of the Cork Work-
house to supply them with a nurse for their fever
department. If, as is doubtless the case, the nurse
works under the supervision of a competent super-
intendent, an opportunity for gaining mo9t excellent
experience is secured to a succession of the workers at
the City of Cork Hospital.
DISTRICT NURSING AT MELBOURNE.
It is now eight years since the District Nursing
Association was first established at Melbourne,
and it is very pleasant to hear of its continued
and ever-inoreasing usefulness. Although the prevail-
ing depression in the Australian colonies has affected
the funds of this, in common with all other societies,
yet it Bhows a very creditable balance-sheet. Three
nurses were employed during the past year, and they
attended 653 patients, paying in all 17,015 visits.
SHORT ITEMS.
The Workhouse Nursing Association expended
?313 in training nurses last year. They have sent
nurses to forty unions, and thirty-three of these sub-
scribe to the fund.?Nurse Durose has been appointed
Parish Nurse for St. Thomas's, at Dudley. The Yicar
is most anxious to secure trained nurses for all the
districts in this town.?Miss E. L. Robertson is giving
a course of lectureB on nursing at Wolston, under the
Warwickshire County Council. It is hoped that before
long a second district nurse will be established at
Berwick. Miss Ethel Lamport's second course of
lectures on sanitation commences this week.
May 6,1893. THE HOSPITAL NURSING SUPPLEMENT. liii
Sanitation for Burses.
By a Sanitary Inspector.
IV.?SWEDEN.
When we come to consider the state of sanitary science in
Sweden and the hygienio regulations there laid down for the
preservation of the health of the inhabitants of that country,
we cannot but be impressed by the perfection of the adminis.
tration. Here we find regulations which in their complete-
ness and stringency more nearly approach those in force in
our own country than any we have hitherto found in the
Continental towns already considered, Moreover, the low
mortality found in this country (lower, we believe, than that
in any other), bears ample testimony to the excellent sanitary
arrangements in force.
The supreme authority in thejcountry is held by the Medical
Council?composed of only four members, one of whom has
the supervision of military and naval sanitary affairs. All
matters which cannot be settled by subordinate sanitary
bodies can be finally decided by this Council, and all reports of
public health, treatment of the sick, &c., are sent in to it. It
ia amusing to note some of the institutions which come under
the control of this Council. In addition to suchobvious ones
as institutions concerned with public hygiene, the care of
the sick, and asylums, we find bathing establishments and
barbers' shops are not beneath the attention of this august
Council. We can only wonder why the line has been drawn
at these, and why the Medical [Council does not extend its
beneficent control to bakeries, laundries and many similar
establishments. The special and peculiar dangers of barbers'
shops and bathing institutions are not quite so clear to us as
they apparently are to.the Scandinavian mind, orperhaps in
Sweden there are special risks which do not exist in other
countries.
In provincial towns the provincial doctor has the direc-
tion of public health matters, his authority being of course
limited to his own immediate province. His duties are very
numerous.and varied, and if carried out to the letter, must
require great organisation. The provincial doctor seems to
mperBonate the different roles of medical man, sanitary
inspector, lecturer on health, and father of a family.
According to Palmberg, he has " to keep a watchful eye over
the welfare of the people, and give attention to everything
likely to affect the health of the people." He is expected to
study the medical topography of his own province, the cha-
racter and manner of life of the population, to inspect rural
dwellings, and have all sanitary defects set.right; he has to
superintend the physical education of the children in the
country, to superintend the various domestic remedies in
Vogue, give instruction as to the proper use and applica-
tion of such, and oppose all excess, and he is expected to
diffuse useful knowledge on sanitary matters in all directions.
-The provincial doctor haa further to see to the quality of the
fooda and drinks consumed, and especially so when " danger
to health may be apprehended after a bad harvest," or should
bad weather prevail during the harvest. All such events
and defects are to be duly reported to the Governor of the
Province, and if necessity should arise ho can apply for funds
to relieve want, and the Governor will further take care that
all necessary preventive measures are taken.
ThiB is no doubt an ideal state of affairs, and if the provinces
be divided up .into sufficiently small districts, and enough
provincial doctors be appointed,{and the people are willing to
submit to their minute inspection and supervision, the Scandi-
^avians will Boon be the longest lived nation on the face of
the earth. We shall hear of no deaths from disease,
hereditary or acquired, the people will Burely die simply of
old age.
In addition to the provinces, we further find eaoh country
district provided with a "Ccmmunal Council," and the large
towns with a " Health Commission." The respective powers
of these are similar, the only difference being that one has to
do with the country and the other with the towns. It ip,
however, a little difficult to distinguish between the duties
of the provincial dootor and of the Communal Council.
The Health Commission consists of a prefident, vice-
president, and about a dozen members, amongst whom i3 the
head of the police. This Commission is really a sort of
sanitary police, which has supervision of sanitary matters,
and rules its district in that respect with a rod of iron. It
works with the municipal police, who report any sanitary
defects which come under its notice. It lays down very
rigorous regulations, which it is compulsory on the people
to carry out, disobedience or neglect being punished by fine.
It nan also Bummon any citizen to attend before it, and if the
order be not complied with, the police compel Buch attend-
ance. In all respects the Commission is thoroughly imbued
with the importance of proper regard to tanitary matters,
and gives no loophole for evasion. We find no " suggestion"
or " advice " offered, only stern uncompromising rules laid
down, and a fine imposed if such be not kept. It is amusing,
therefore, to find that there is one person who can defy
the Commission?that is the chief of the police. With him
rests the carrying out of any sanitary ordinances set forth
by the Commission, but if he considers these are not in
accordance with any general public regulations he can refuse
to carry them out, and can then refer the matter to the
higher authority vested in the Royal Prefecture. The Com-
mission makes careful inquiries into the causes when an
increase in the death - rate is reported from any
part, and, in the case of an epidemic occurring,
has the power to absolutely forbid the sale of
unripe fruit, or of any kind of fruit deemed hurtful
under the circumstances, and for the time being, in that par-
ticular district. One special department of health is under-
taken by the Commission, and we wish all countries would
follow its example?viz., the due ventilation of all public
buildings, not only schools, factories, workshops, and work-
houses, but even churches, law-courts, and theatres, down
even to restaurants and hotels.
In the capital, Stockholm, a Bureau of Health superintends
all sanitary matters, and is under the direction of the chief
doctor in the city. An inspector and a doctor, called the
" Head of the Sanitary Police," are attached. There is one
Btrange anomaly in the otherwise excellent hygenic regula-
tions, and this is, that all authority is vested in the Bureau,
with the Bingle exception of the supervision and right of in-
spection of the sanitary arrangements in private houses, which
belongs to a special Commission appointed for this sole
purpose. This division of authority often leads to most un-
necessary friction.
It is almost needless to say that notification of all infectious
diseases is compulsory, so also is the removal of the patient
to the hospital, unless either such removal is certified by the
sanitary authority to be likely to be hurtful, or unleBs it is
proved to the satisfaction of the authority that the patient
can be properly isolated and nurBed at home.
In 1890 a special isolation hospital for infectious diseases
was begun. It consists of five pavilions, in which are nursed
respectively scarlet fever, typhus, measles, small-pox, and
diphtheria. It contains 162 bedB, and in addition a separate
building with eight observation rooms.
The regulations regarding schools are very thorough, the
medical examination of the children and the sanitary inspec-
tion of the buildings being carried out at regular intervals.
In case, however, of infeotiouB disease breaking out, the
decision whether or no the school Bhall be cloBed reBts with
the Bchool authorities, and not with the supreme sanitary
authority, as is usually the case.
liv THE HOSPITAL NURSING SUPPLEMENT, May 6, 1893.
B German Ibospttal.
Br An English Patient.
II.?DOCTOR AND DINNER.
The dootor may be expected any time after breakfast. As
soon as he is seen to leave the neighbouring ward, the nurse's
loud exclamation, "The doctor is coming!" reduces the
children to silence. The convalescents betake themselves
each to the foot of her bed, and by the time he appears
all is in " apple pie " order.
All the doctors seem to wear eye-glasses, and to bear upon
their faces the marks of valorous achievements in their
student days, and arrayed in long white coats, adorned with
large metal buttons, they strongly resemble highly respect-
able coachmen. The Germans are very fond of a uniform,
especially if it be well sprinkled with bright buttons. The
doctor is rarely in the ward more than five minutes, includ-
ing the time spent in writing prescriptions, so the little ex-
citement is soon over.
Oar doctor always spoke English with me, a source of
vexation to the nurse, who was curiosity personified,
and always paid the greatest attention to what he
Baid, hoping to understand something. One evening he
sat down on the edge of the next bed while talking to me.
She stood behind, shaking her fist at me in mock anger, why,
I had not the ghoBt of an idea. I heard afterwards that she
had some potatoes frying for her supper, and that the doctor
should take a seat as if he were going to talk for half an hour,
instead of making his visit with his usual speed, was too
aggravating, and the potatoes burning in the meantime !
This particular doctor was in England for some years, and
on his appearance at the hospital the nurses declare they were
disgusted at what they considered his English brusqueness
and impatience. In course of time he has toned down and
greatly improved in the eyes of the nurses, and now they all
" schwarmen" for him.
About once in a week or ten days we are greatly exercised
in our minds by the sudden news that the head doctor (Herr
Professor) with his suite of attendant doctors and nurses,
has been seen in the neighbourhood, or perhaps even in the
next ward, when we know for a certainty that he will be in
upon us in two minutes. It is rather alarming to Bee four
long white coats appearing in the doorway, closely followed
by an over-nurse and an inspector hovering in the rear, and
still more embarrassing when the wearerslof these long white
coats draw up in line in front of you. Herr Professor does
not pay much]attention to any but serious cases.
After the doctor's departure, speculations are raised as to
what the fprospective dinner, which is Berved about two
o'clock, will consist of. The clanking of the huge cans (or
buckets) in which the dinner is brought iB always a welcome
sound, for, even if the dinner is not quite ao recherche as one
could wish, meal times do break the monotony of one's
existence.
German invalid fare is peculiar, to say the least of it.
Such delightful things as beef tea, bread and milk, and toast
are unknown; indeed, the latter is not to be had, as there are
no open fireplaces. There are four different forms of diet; for
the midday meal the first is milk, and milk soup ; the second,
plenty of mashed potato, a little meat, and milk soup if de-
sired ; the third is Bimilar to the second with the addition
of meat soup; the fourth form consists of a variety of things,
and is for patients who are pronounced well, and are only re-
maining in the hospital for safety's sake. Those who have
first form diet do not get any breakfast at ten o' clock
which is rather hard lines, especially when they do not feel
at all ill,
I do not wish to depreciate the diet; nothing is unfit for
food, but I must say that it would be better if considerably
less food was sent, and if it was better prepared. It is not
very badly cooked, but there is too much economy practised.
The patients in this ward are all servants, and not too
fastidious as to what th?y eat; yet often the meat is hardly
touohed, and every day about a gallon of soup, and much
else besides, is wasted. Everything that remains over from
dinner is thrown away ; the soup is disposed of in the ward,
and other things are thrown together into a basket and carried
away to the pigs.
I will try and describe, as accurately as possible, one
dinner: Meat soup, a thin rather watery soup with
vegetables in it, milk soup, rather watery also, mashed
potato, meat, rice, boiled in milk and water, with a sprink-
ling of raisins and cabbage, or what was so-called, potato
being by far the predominating element. Vegetables never
appear except in combination with potato or in soup. Some-
times there comes a kind of thin porridge which, when well
made, is much better than our oatmeal porridge.
The little ones have children's diet. Meat about three
times a week, and on other days pancake (German) or
pudding, and, of course, every day soup, either made of fruit
or meat. It is unanimously agreed that our ten o'clook
breakfast is preferable to our dinner.
After dinner the under-nurse, assisted by one or more of
the convalescents, clears away and washes the dinner things.
All vessels, for whatever purpose required, are kept in the
ward, and mustsbe kept clean. Two hours of comparative
quieb follow, during which many of us go to sleep. The beds
are not allowed to be used in the daytime unless one com-
pletely undreises ; and our day-room not being luxuriously
fitted up, I usually ensconce myself on the operating table,
where the only thing which disturbs my slumbers, is the fear
of falling off on one side or the other, for the table is very
narrow. At four o'clock] wa gather round the huge coffee,
can.
This is a peculiarly German meal, consisting simply of
coffee with bread and butter and coffee-bread. Eaoh child
receives with her coffee one thick piece of bread and a piece
of c#ffee-bread ; the latter being merely slices of white bread
cut rather thick and baked until there is not a particle of
moisture left in it, and it is quite crisp throughout. It is
rather nice, and is the nearest approach tojtoast that I have
ever tasted in Germany.
Every Saturday, after]'coffee time, we have to don our
night-gowns and be weighed, and the amount each patient
gains or loses is the criterion by which the " pillars of the
establishment" judge whether the diet is beneficial or not.
The next event is the doctor's evening visit, which may be
expected any time before six, or even earlier, [if he is going
out in the evening. The ward is lighted by electricity; at
the best it is only a " dim religious light," insufficient for the
doctor to examine anyone; so if he comes late, the nurse
follows him cloBely, carrying an electric ,light in her hand,
which is connected by a long rope to the eleotricity above,
and by this meanB each bed is illuminated.
The appearance of a new doctor always creates a diversion.
One evening our usual Esculapius was unable to come (at
least, we suppose he was?he surely would not willingly
desert us !), and there entered in his stead a ponderous man,
in fact, lying in bed, as I was at that time, I could not take
him in at a glance. Something which looked like the handle
of a big knife protruded from the pocket of his linen coat, so
that ho really looked like a great butcher amongst the
nurses; he goes by the name of " der Schlachter." Well,
this "big gun marched in, planted himself in the middle
of the ward, turned round three [times, ^ and tramped out at
the other end !
The last event rof the day is supper, at half-past seven,
consisting of bread and butter, and at nine o'clock the day is
ended and everyone, nurses included, must retire to bed. The
Mat 6, 1893. THE H0SPI7AL NURSING SUPPLEMENT. \v
head nurse has her own room, but the under-nurse sleeps in
the ward, and is supposed to attend to the wants of the pa-
tientB during the night. She sleeps so soundly, however,
that generally one of the convalescents is up and has satisfied
the wants of a crying child long before Bhe thinks of awaking.
The lights are turned out about nine o'clock, with the excep-
tion of one whioh faintly glimmers until daylight, when it
extinguishes itself.
My first impression of tho German hospital bed was that
it waB ingeniously contrived to prevent one feeling any com-
fort therein. The bedstead is of wood and iron. There is a
hard mattress, under which is a spring mattress, which does
not merit its name. There is a wedge-shaped horsehair pillow
or bolster, over which comes the sheet, then another horsehair
pillow for the head, and lastly the cover. On a nice German
bed, this c?ver would ba a beautifully light feather bed, but
in hospital it consisted of a thick blanket folded twice and
put into a linen bag. It is said that a German must live seven
yearB in England ere he ltarns how to stir a fire with the
requisite dexterity ; one can say with as much truth, that an
Englishman must serve as long an apprenticeship to sleeping
in a German bed, ere he can keep his cover on all night. There
is no possibility of tucking it in at the sides, or foot, the con-
sequence is, one is liable to wake in the smallest hours of the
morning to find the wretched thing all but gone, and frantic
efforts may result in landing it " all of a heap " on the bed
again. Such has been my experience. About three times
during the night a nurEe walks through the ward to see that
all is right.
(To be continued.)
District IRurses.
Districts vary as much as nurses, there can be no doubt
about that. In London and other large towns the popula-
tion in the poor neighbourhoods is a shifting one. The
families move suddenly and silently, leaving very often no
clue to their new domiciles. Therefore, the work amongst
London poor is an altogether different business for the nurse
than in country districts, where the same people inhabit the
same cottages for many successive years. In some places in
the North of England, for instance, where wages are good,
and the people help one another, there is neither urgent
poverty nor any great demand for extraneous help in sickness.
Bat because persons are willing to aid each other it does nob
prove their competency, and, therefore, skilled assistance and
instruction in home-nursing are beginning to be highly valued
hy the self-reliant and kindly North-country folks.
But as they are naturally ahrewd and intelligent, the
teaching muBt be good to be acceptable. Until they feel con-
fidence in the trained attendant they will not leave their sick
friends in her hands. In other country districta again,
nurses find the neighbours most willing to be helpful, and a
little management with regard to the services accepted will
ensure sufficient provision for the constant care of the Bick.
It ia necessary to insist on economy in the number of
watchers, otherwise the tendency for a group of neighbours
to remain on the alert for a night or two is succeeded, when
the case grows monotonous, by the conspicuous absence of all
the friends.
It is much harder in large towns to find competent friends
to share the nurse's duties methodically. There is not the
same friendly intercourse in the densely-populated streets
as there is in country placeB, and the pressing call of deep
poverty necessitates all women who can earn anything, doing
8o> to the neglect of near demands of charity.
One nurse of large and practical experience has made a
novel but most satisfactory experiment. She found that
when she visited a sick person regularly, the other members
of the household quickly got into the habit of relying upon
"nurse's bag" for every needful comfort. This was not
from any unworthy motive, for the people were neither
greedy nor very poor, but the materials which came out cf
that well-known receptacle appeared always better and
more suitable than their own, and] they|were shy of volun-
teering the latter.
The nurse was a wise woman, and she saw the impending
result. Instead of increasing the self-helpfulness of those-
she was longing to serve, she was gradually diminishing
their reliance on their own resources. She put away her
bag. She went into the sick rooms empty handed, and in a.
marvellously quick time she had influenced those observant
country women to anticipate her wants. If the best thing
for any purpose was not immediately available, she showed
them how to utilise the second besi as a substitute. They
took a pleasure and pride in displaying their ingenuity, with
the result that the patients largely benefited in process of-
time.
Hence tact in the teacher is as necessary as knowledge, and
perhaps this applies to sick nursing more than to any other
pursuit.
In some places the district nurses are asked to " lay out
or "lay away" the dead, but this is not usually the rule.
In the North very often months, nay years, before death,,
people have calmly decided^by whose handB they wish the
last offices to be performed. There is a simplicity not with-
out dignity in such arrangements as these, and it does away
with the professional harpy so well portrayed by Dickens.
It seems to give a kind of moral support to a!person " sick
unto death " to know that these details are finally settled.
Probably it has the same kind of influence as the knowledge-
of having "made his will" exorcises on a man of a richer
class.
And the district nurse has usually so many duties to per-
form for the living that she cannot linger long beside the
dead; therefore, it is more becoming to leave such tazks a&
she can, to the hands of the surviving relations.
Beyond and above being a thoroughly efficient nurse, a.
district worker needs to be a handy and practical woman.
She has to rely solely upon her own discretion in many
sudden emergencies. She needs to be ready in speech as in-
action, bright and cheerful, giving and deserving confidence.
Moreover she ought to possess pe rfect health. This i6?
really essential for a person who has a great deal of walking
to do, constant changes of weather to face, and frequently
to content herself with hurried and delayed meals.
Even after starting the work in a perfectly satisfactory
state of health, she may break down under undue pressure
or from the atmospheric trials to which she is exposed When
her illness is contracted in the discharge of her duty, she
certainly has a claim on her employers, and they ought to-
feel it her simple right to be cared for and nursed back to
health at their personal cost. That is to say, she ought to-
have medical attendance and such additional nourishment ae
the doctor deems necessary, supplied to her freely and
willingly.
We wish this were an invariable rule in all local nursing
institutions, for it certainly Beems hard that the decision
should lie in the power of one or two unpractical ladies or
gentlemen who are quite undecided whether to acquiesco
cheerfully in granting proper nourishment for the nurse who-
has served the sick poor faithfully, or if they shall simply
dole out the grudged "charity" with a hint that it is by
their own capricious goodwill that this benefit is granted ?
Justice should be done to the worker, as well as to thoss
she is paid to work for. The question lies in a nutshell, and
a fair right should never be merged in a favour.
In their work district nurses may advantageously aim as.
much at teaching as at doing. The latter is much easier
than the former to most people, but it is not nearly so help-
ful. The nurse cannot always be present, and therefore
the greater the aid she can train the patient's friends to-
give her the better for all concerned. In this way her own.
experience is utilised to the utmost possible limits.
lvi THE HOSPITAL NURSING SUPPLEMENT. May 6, 1893.
St. flDaro's, flMaiatow.
OPENING OF THE NEW DAY NURSERY.
?ScRELYjJf any parish needB a big Day Nursery, St. Mary's
does. The district is so large and so uniformly poor that
the mothers generally have to " go out to work," and even
then the family's earnings are very scanty. Hence the
babies have a bad time of it in every way, and a well-
managed day nurBery on a large scale is just the very best
help that can be given to these small folks. A large party
assembled on Saturday afternoon, the 29th ult., to witness
the opening of the newly-built Day Nursery by the Duchess
of Westminster. After a short service of prayer and praise,
the Vicar (the Rev. Given-Wilson), in a few well-chosen
words, addressed the meeting, referring in grateful termB to
the gift of ?3,800 by an anonymous donor. The building
will accommodate 150 babies, and is vested in Bix trustees,
viz., the Bishop, the Archdeacon, and the Yicar of the
Diocese, with three laymen. He closed by earnestly thank-
ing the Duke and [Duchess of Westminster for their many
?cts of kindness in connection with the work, especially the
laying of the foundation-stone of that building by her Grace.
"The Duchess made a short speech, and expressed her plea-
sure in being present, and declared the Nursery open. The
Duke of Westminster remarked that he had gladly availed
himself of this opportunity for assisting at the opening of the
largest day nursery in the world. He drew attention to the
good work in which Mr. and Mrs. Given-Wilson were engaged,
and reminded those {present that ponies, bath chairs, tea,
sugar, &o., would always be acceptable presents to the insti-
tution. He concluded by wishing Mr. and Mrs. Given-Wilson
every success in their philanthropic work. After short speeches
by Canon Proctor, Canon Stephens, the Rev. R. A. Pelly,
the Lord Mayor, and Mr. David Howard, J.P., the benedic-
tion was pronounced.
The Duchess then repaired to the Nursery to choose a cot
to bear the name of her youngest daughter, "The Lady
Helen Francis Grosvenor." This cot will be specially
reserved for stray waifs such as the nurses occasionally pick
up on their rounds.
The swinging cots, each containing a contented looking
baby dressed in scarlet, were eagerly inspected by the
visitors, and the bath-rooms were much admired, so suitable
did they seem for the requirements of the small people.
Well-furnished linen cupboards with glass doors were as
pleasant a sight as the play-rooms, rich in swings, and rocking-
horsaa. Pegs, and bags to contain the clothes in which the
'babies arrive, and in which they are sent home again in the
evening, are also kept in orderly array.
The proceedings terminated with a fancy fair. Among the
stalls specially deserving of notice was that of Miss Given-
Wilson and Miss Cunninghame, who wore B.A. caps and
gowns. Miss Fitch and Miss T. Given-Wilson, as flower
.girls, presided over a stall laden with lovely blossoms. Miss
Fitch and Miss Williams, in charming milkmaid costumes,
dispensed butter, eggs, and cream. Miss Heinekey, as a
?Quakeress, and Miss lies, Spanish maiden, sold wholesome-
looking bread and cakes; whilst a young coster in mar-
vellous buttons and velveteens cordially invited all present
to have their fortunes told. We hope the funds will prove
to have substantially benefited by the proceeds of this well-
arranged entertainment.
Wants an& Morftcrs.
Wasted a Home for an aged female who is blind. Six shillings a
?week could be paid.
Nuese O. and her patient wish to thank the editor and other* for the
many kind replies sent in answer to the inquiry inserted in this column
lXor a home for an invalid clergyman.
JEven>bot>?'s ?pinion.
[Correspondence on all subjects is invited, but we cannot in any way
be responsible for the opinions expressed, by our correspondents. No
communications can be entertained if the name and address of the
correspondent is not given, or unless one side of the paper only be
written on-] ???
NURSE SNORERS AND OTHERS.
" C. M. A." writes : In your issue of The Hospital, April
22nd, I noticed that Dr. Rand, of New York, had, in the case
of a poor man dying of apoplexy, given instantaneous relief
to his stertorous breathing by " pressing upwards and for-
ward under his chin. A cardboard prop from the chest
was therefore improvised, the flesh being protected by a
handkerchief ; it was easily kept in position by the nurse,
and throughout the twelve hours which the patient survived
the respiration continued quiet and natural." Now, could
not something of the sort be used by persons who snore so
inveterately that they become a nuisance to everybody in
the house ? Especially should it be recommended to nurses
who unfortunately are frequently afflicted in that way. I
have frequently heard patients complain that they were
unable to sleep on account of their attendant's snoriDg.
A friend of mine was isolated for six weeks last
summer with scarlet fever, which affected her heart. Every
night, when the palpitation ceased, and she could have
dropped off into slumber, the noise of a trombone, played
with much vigour, began, and continued with but slight in-
termission through the night, till the poor young lady
became so nervous and distracted that she wept away the
time, being too weak to stop the performance. This is by no
means a singular case, and if a cure for snoring could be
found, the discoverer would be among the great benefactors
of humanity.
THE LIFTING OF PATIENTS.
" A Male Nurse " writes : The lifting of patients is to'some
nurses a serious question ,when left single-handed, especially
when the patient is extra heavy and the bed high; the lower
the bed of course the easier the task. Now to the majority of
nurBes the lifting of a patient without injury is a difficulty,
especially when he is helpless and lies like a log of wood.
How can a Bhort nurse raise a helpless person on a high bed,
and most private cases are found on beds exceptionally
high and wide ? It is well-nigh impossible, especially when
the lifting has to be done from the side. When I am in this
predicament, without help at hand, my mode is this : I take
my shoes off, mount the bed, place one leg each side of the
patient, so as to have him directly under me, then I place my
arms under his, lace my fingers together, and lift in this
manner a very heavy man with ease. If the back of the
patient is tender, substitute a towel for the hands. I have
never since I adopted this method had the slightest difficulty
in lifting the heaviest of my patients. It is easy for both
nurse and patient, and the weak need fear no strain. The
practical simplicity of this plan will commend it to sensible
persons after they have once tried it.
#** We are always glad when male nurses make sugges-
tions in this column, but our present correspondent only
deals with one special form of "lifting," viz., raising the
patient higher up in bed. The article which we recently
published on the subject dealt with a wider aspect of
the subject.?Ed. T. H.
Marriage.
Miss Susan Barraud, late Matron of the West Quay
Hospital, Southampton, was recently married to Dr. Arthur
Wellesley Harris, Medical Officer of Health and Port
Sanitary Officer for the same town.
Mat 6, 1893. THE HOSPITAL NURSING SUPPLEMENT. Ivii
(Stuarantine tor IRurses.
When a nurse attached to a hospital has been sent out to
any case of infectious disease, she finds on her return that
all the responsibilities of quarantine, &c., are provided for
by the rules of the institution. But a large number of
nurses are engaged in private work, and many difficulties
beset the path of some ofjjthem.
Nursing institutions-which supply nurses to the public,
ought to have special arrangements to meet the case, and, of
course, many of them have adequate accommodation for it.
But there are numbers of nurses who find themselves in a
difficult position on leaving patients who have had measles,
scarlet fever, &c. Of course, they go through a full and
satisfactory process of disinfecting their own persons and
fumigating their clothes at the conclusion of their engage-
ment, and are in every way bound to make themselves abso-
lutely safe to mix with their fellows. Practically there is
but one reason why they should not do this, and that is the
pcssibility that they have contracted the disease with which
they have been associated. It is, therefore, quite necessary
that they BhouJd avoid the society of other nurses, children,
and delicate persons, during the full period of incubation.
Very few employers of the private nurse give a thought as
to what becomes of her after they have paid her fee and said
good-bye.
With regard to the fees, too, a word may be said, for they
are rightly enough fixed at a higher rate than the charge
for attendance on non-infectious illnesses, but the extra earn-
ings do not cover the extra expense to which the nurse is
put, unless the patient needs her services for several weekB.
For instance, in measles, when the nurse may be only
wanted for a week or a fortnight, she has afterwards to
undergo a fortnight of quarantine, and the extra guinea she
has earned will not cover her expenses. If she lives at a
nurses' club, or in a house where there are children, it is need-
ful for her to go elsewhere. This means paying a double
rent, although the cost of board will be probably only the
same. Now where does she spend her " quarantine " ?
Doubtless some private nurses have friends who are
willing to take them in under all circumstances, but there is
Btill an opening for special accommodation where workers
will be welcomed, not shunned, at the conclusion of their
attendance on infectious diseases. We hear of a recently
opened Home for Quarantine Nurses, at Upper Holloway,
of which the address was given last week in answer to "Query"
1*1 o. (112), and we Bhall be glad if any of our readers will tell
us of any seaside home for this purpose of which they have
personal knowledge.
appointments.
Grantham Hospital.?Miss Elizabeth Shannon has been
appointed Matron of Grantham Hospital. Miss Shannon
was trained at Glasgow Western Infirmary, and afterwards
held the post of Assistant Matron at the Stanley Infirmary,
Liverpool.
Guildford Infectious Hospital.?Mrs. S. Chambers has
been appointed Matron of the Guildford Infectious Hospital.
Mrs. Chambers has proved herself so able an administrator
at the Wolverhampton Borough Hospital, that the Guildford
Committee are to bo congratulated on having secured the
services of this efficient matron.
Bromsgrove Cottage Hospital. ? Miss Eveline J.
Worthington has been appointed Matron of the Bromsgrove
Cottage Hospital. Miss Worthington was trained at the
Suseex County Hospital and Children's Home, Brondesbury,
and has had great experience in medical and surgical nursing.
For the last four years Mies Worthington has been in charge
of Andover Cottage Hospital.
Swansea General Hospital.?Miss Sykes has been ap-
pointed Matron of the Swansea Hospital, where for the past
two yearB she has held the post of sister. Miss Sykes
performed her duties in the latter capacity in such an
efficient manner that she appears to be in all respects well
fitted to hold the more responsible office to which she has
been recently nominated. Misa Sykes was trained at the
General Infirmary, Leeds, and afterwards worked at the
Women and Children's Hospital in that town.
jfor lRea&ing to tbe Stclt.
PRESCRIPTIONS. ?III.
We have been thinking lately about the prescriptions which
God eees fit to order in His good providence for the health of
our souls ; how he sends us, not all bitters and acids, nor all
sweets, but just such a mixture as will cure us if we will only
accept the remedy and apply it carefully.
It seems sometimes very difficult to do this, but the
Almighty has not left us without full instructions, and they
are written in the Bible for our especial benefit. For instance,
sickness is sent to curb our independence, to show us how
weak and powerless we are without our Heavenly Father's
care, that if He takes away His breath we die and are turned
again into dust. To reconcile us to this we read, " Whom
the Lord Ioveth He chasteneth," let us then rejoice in His
love. If frebfulness and irritation come in affliction's train,
we must not give way to them lest they increase, and our
Lord's words of counsel in this instance are "in patience
possess ye your bouIs. '' Ah 1 if we can only have a small
portion of that grace, by which Christ bore the cruel
scourgings and buffetings of His enemies, the hunger, thirst,
and sufferings of His life and cruel death, we may smile in
trouble and kiss the hand which holds the rod,
saying with the Psalmist, " Thy rod and Thy staff
comfort roe." Can troubles ever comfort us ? Yes, because
they assure us that we are thought worthy to tread in His
footsteps and carry the cross a little while with Him. A
very sharp and troublesome complaint c?n be dealt with
successfully by taking the remedies mentioned in many parts
of the Divine book. We read that tbe tongue setteth on fire
the course of nature, that it is an unruly evil very difficult to
tame, and so we must keep watch over ourselves if we are
inclined to err in that way, remembering that " he who
can rule himself is better than he who taketh a city," while
the bitterness of our neighbours' tongues can be met by the
soft answer which turneth away wrath. In fact, every ill is
lessened by a strong dose of patience, but how are we to get
it, where shall we find it ? By going to the Great Physician,
because He says " Ask and it shall be given unto you, Beek
and ye shall find," and " Whatsoever ye ask the Father in
My name He will give it you." Prayer moves the Arm
which rules the world, therefore let us pray without ceasing,
we need not be always on our knees, but our hearts may be
bent to ask for help at any moment. " More things are
wrought by prayer than this world dreams of," says the
Christian poet, " wherefore let thy voice rise like a fountain
night and day," and he goes on to remind us, men are no
better than sheep and goats if knowing God they do not lift
up their hands in prayer, both for themselves and their
friends.
Habere to (So.
Hampstead Home Hospital and Nursino Institute.?
A fancy sale will be held at the hospital on Friday and
Saturday, May 12th and 13th, followed by a Rummage Sale
on May 20th. The hospital is close to Hampstead Heath
Station.
Steinway Hall, Lower Seymour Street.?A grand
evening concert, under the patronage of the Duchess of
Westminster, will be held on May 15th, at eight p.m., in
aid of the funds of St. Mary's Home, Plaistow. Tickets to
be obtained of Miss Pritchard, 41, Cleveland Square, W.
On Wednesday, May 10th, at eight o'clock, a grand
evening concert will be given at St. James's Hall. A number
of well-known artists will assist, and Miss Nora Hasting, by
whom the concert is arranged, will give some dramatic
recitals.
Another grand evening concert will take place at St.
James's Hall on Monday, May 15th, at eight o'clock, in aid
of the Middlesex Hospital Chapel Fund. The concert, which
is given by Mr. Otto Cantor, is under the pantronage of the
Princess Christian and the Duke of Cambridge.
lviii THE HOSPITAL NURSING SUPPLEMENT. May 6,1893.
Iber better Self.
III.?" FAR ABOVE RUBIES."
When old Captain Reece next awoke he was considerably
surprised. It was somewhere about the dawn of a cold April
morning, for Daphne had been gone two months, but he was
not in his iron bedstead, nor yet in the smoke-room. He
was lying on the wet grass lawn; a gesticulating, excited
crowd around him, a confused roaring and hissing in his
ears.
" He'll do now !" said a cheery voice, which the captain
could have sworn belonged to little Grigsby, that young
whipper-snapper of a new doctor who had come to the village.
" I wish to goodness I could say the same of poor Dunsford,"
continued the speaker.
" He be tarrible brunt, I expec'? " hazarded another voice.
Then the captain opened his eyes, and grasped the situation.
There had been a fire ; the dull roar, and the hiss of water
fighting with its tigerish foe, were evidences enough.
" I'm still living in my tkin," muttered he, after cautiously
feeling himself all over, "it's not burnt off me. I wonder
why I'm lying here? I must get up and do something."
All he did, however, was to slip baok into a swoon ; the
old man had been nearly asphyxiated, and only rescued in
the nick of time.
Just before dawn a farm hand, who had been up all night
with a sick cow, rushed to his master's room, shouting in an
exhilarating tone, " T' Bunk's afoire, sir ! " and Harry had
been on the spot almost before he understood the summons.
With reckless dash he entered the burning building, to find,
among the dense, rolling volumes of smoke, the unconscious
captain, whose pipe, setting fire to a waste-paper basket, had
brought about the catastrophe. In shielding his senseless
burden from the flames as he bore it out, Harry got grievously
burned about the face. In the days that followed little Dr.
Grigsby had his hands pretty full; but the tough old sailor,
unscathed from the fire, was the first of his patients to
recover.
"We mustn't let Daphy know," insisted the captain;
" Can't have my girl terrified."
"No, Daphy mustn't know,'' echoed somebody else
chokingly ; and soon after a broken.down man groped about
with outstretched hands, feeling, not seeing his way, for
Harry Dunsford's bravery had cost him his eyesight.
Meantime, there were charges in town also. Daphne was
turning out a dire disappointment, to Aunt Sarah's dismay.
Ever since the day when a chance face, a pure steadfast face
iu the crowd, set Daphne thinking, she had been trying to
discover how she, too, might help on the wheel that needs so
many shoulders to keep it going. There's no place like
London, she Boon knew, for opportunities to help one's
fellow creatures ; eagerly she rushed into the thick of that
never-won battle with the sighing and sorrowing of the poor.
That was Daphne all over. Steeped to her laughing lips in
the season's delights she turned round, fain to descend to
the depths of the anguish behind, that accentuates the
gaiety and glitter of London pleasures.
"I wash my hands of you," said Aunt Sarah grandly;
then she broke down and wept, thinking to win back
Diphne.
"No," decided the girl, "my eyes have been opened; I
aee that I wasn't sent into this world to
' Laugh and quaff,
And dance and chaff,'
for that's your life, Aunt Sirah, say what you will.''
* * ? * *
' Oh Dad, what joy to be back in the clean country," cried
Daphne, hugging the excited old captain, who proceeded to-
pour out his hoarded up tale of the recent " peril by fire,"'
enjoying the sensational effect he produced. But when
Daphne heard of the cost of her father's life she was stunned.
" Yes, he's blind, poor chap ; can't see his way about. I
feel ashamed to ba alive, my dear, when I look at him ! "
But Daphne was too choked to answer. Was the bright
world blotted out for Harry ? Would ha never again see?
herself ? At the latter thought a sword pierced her heart.
"Take me to him?to my Harry !" she wailed.
" Bless my life, child ! " Then something the old captain
saw in the set, white face told him a volume. "Daphy,1*
he went on quite softly, "is that ib ? My dear, come, I'll
tike you to the poor chap."
An hour later, a girl was kneeling by Harry Dunsford's
side weeping her heart out over his folded hands?the hand*
that steadily refrained from so much as touching her. Ib
was a maddening moment; the stricken man groaned in th?
anguish of feeling Daphne so near, and knowing how far?
further than ever?she was from him.
" Who takes care of you, Harry ? " she sobbed out, when
her passion of grief exhausted itself.
"Oh?nobody!" jerked out Harry abruptly. The situa-
tion was getting more than man could bear.
"Will you let?me?" Was it Daphne who whispered
the bold question ? Impossible ! Still, the shamefaced girl
waB hiding her blushes, from the eyes that could not see them,
on Harry's shoulder.
" That's what spoilt the girl's chance ! " ejaculated Aunt?
Sarah, when she heard the news of Daphne's engagement.
" A miserable country entanglement ! Disgraceful ! "
'?Yes, 'm," sympathised Masters; "and to think she
might have married a kerridge and-pair ! "
But Aunt Sarah was wrong. It was Daphne's better self
that, called forth by the stray vision of a true, tender,,
earnest face in the crowd, made of her the happiest wife in
all the neighbourhood, and a woman, likewise, whose price-
is " far above rubies."
IRotes an& ?ueries.
SPECIAL NOTICE.
The contents of the Editor's Letter-box have now reached moh un-
wieldy proportions that it has become necessary to establish a hard and'
fast rule regarding Answers to Correspondents. In future, all quoBtiona
requiring replies will oontinue to In answered in this column without
any fee. If an answer is required by letter, a fee of half-a-crown must-
be enclosed with the note containing the enquiry. We are always,
pleased to help our numerous correspondents to the fullest extent, and
we oan trust them to sympathise in the overwhelming amount of
writing which makes the new rules a necessity;
Queries.
(119) Training.?Where on I get gsod training as male nurse,.
Medical or mentil??b\ Davies.
(120) Vaccination.?Is it considered essential to be ravaccinated mors-
than once ?- Nur e A.
(121) Co operation.?Is there any co operation of nurses in Glafgow.
M. A. C.
(122) Children's Hospitals.?I shall be much obliged if yoi will t?ive
ma some addresses of children's hospitals in or near London ?? B. Y.
(123) Eorly Vhthisis.? ExDlatiation of symtoms wanted by Marty.
(121) Q.V.J.I.?Can yon tell me how a district can became affiliated
with the Institute ??Sandy.
Answers.
(119) Training (F. Davies).-Apply to the Medical Superintendent,
Berry Wood. Nnthampton.
(120) Vaccination (Nurse A.).?Certainly, twice, and every six or seven
years if you are liable to be in contact with small-pox.
(121) Co-operation (M. A. C.) ?Yes, a Nurses' Association is in course
of formation for Glasgow and the West of Scotland.
(122) Children's Hopitals (E. F.1,?There are many of them, see
" Bardett's Hojp'tal Annual" (428, Strand, W.C.),- Children's-
Hospital, Great Ormond Street, W.C.; East Londcn Children's
Hospital, Shadwell: Norti-Eastern Children's Hospifl, Shoreditch^
Victoria Hospital, Chelsea ; Ta'decote House Children's Hospital at
Bu'hey He?tb; and Miss LobVs Children's Heme at Linghton.
(12 <) Early Phthisis (A/an ).?You had better consult p doctor.
(124) Q V J.I.-(Sandv).?Write to the Superintendent. Q V. J. I., St-
Kat^arine's Hospital, ItJgent's Park,
May G, 1893. THE HOSPITAL NURSING SUPPLEMENT. lix
<Tbe :?ooft Morlb for Women anD IRurses.
[We invite Correspondence, Criticism, Enquiries, and No+e> on Books likely to interest, Women and Nurses, Addre:s, Editor, The Hospital
(?Nnrsss' Book World), 428, Strand, W.O/l
Primer of Domestic Economy. By Edith A. Barnett
and H. C. O'Neill. (London : Macmillan and Co.)
The authors of ' Our Nurses and the Work They Have
To Do" have produced a very Email, but very compact,
primer of domestic economy. This is a subject which is so
much a matter of detail, and detail varying in every district
and in every family, that it is difficult to lay down any
general principle on which to base it; but Miss Barnett and
Miss O'Neill have indicated very well the ideas that should
guide us in choosing a house, buying and preparing food,
selecting clothing, and saving and investing money. We differ
from them, however, in thinking that laundry work should
be given out. Not only is it cheaper at the moment to do
the washing at home, but the clothes are as a rule more
thoroughly and carefully washed, for in most laundries too
little attention is given to the cleansing of clothes and too
much to the comparatively unnecessary starching and
ironing. Moreover, in many laundries chemicals?tabooed ?n
?very well-ordered household?are employed, and in alt there
is a tendency to make too much use of rubbing-boards and
brushes, the result being that garments are worn out before
their time, even if?which is not always the case?they are
ao thoroughly washed that the "clean" clothes which we
put on are not] in almost as insanitary a condition as those we
take off. We think, too, that the authors deal rather hardly
with their sex in expecting a wife to clothe herself and four
children on ?25 a year, while the husband ia allowed the
same sum for his clothes and " personal expenses." The wife
is, apparently, to have no personal expenses ; but do our
authors know how fast healthy children wear out their
clothes ?
The Emancipation of Women. By AdkleCrep^z.
This little volume has attracted some attention in its
translated form in this country from the circumstance that
Mr. Gladstone has pronounced it in a letter to the authoress
to be " by far the most comprehensive, luminous, and pene-
trating work on this question" with which he has met.
Now, in traversing this rather dreary, dusty, and over-
trodden ground, Miss Crepaz appears to diverge but little
from many of her predecessors. But in upholding the
sanctity of motherhood and the paramount claims of family
life upon women, she is doing better service than if she had
hunted for new conclusions with a strained eagerness after
originality. It is not, perhaps, altogether fair to assume as she
does that the more fully a woman's powers are developed the
weaker becomes the maternal instinct. The evidence points
all the other way. Have we not the testimony of the chief
woman of the century, who in the height of her success aB
an authoress wrote to Leigh Hunt on her little boy's illness.
"You are aware that of that child I am more proud
than of twenty Auroras, even after Leigh Hunt
has praised them." It is the mothers whose minds
are stunted, and whose interests are narrowed,
lx THE HOSPITAL NURSING SUPPLEMENT. may 6,1893.
THE BOOK WORLD FOR WOMEN AND NURSES-coniinwei.
who are apt to find their children dull also, and to escape
from them whenever possible to the distractions of dress or
gossip. Miss Crepaz writes: " There are married lady
doctors, too, and we cannot understand how they can guard
against their professional claims warring with their home
duties. A German authoress tells us that out of thirty-four
married lady doctors practising in New York, there are only
two who have not nursed their infants themselves. It sounds
almost incredible that amid all the suffering and discomfort
of motherhood and while nursing her infant, a mother should
be able to carry on her practice as doctor; going up and down
the many stairs, breathing the unwholesome atmosphere of tbe
sick-room, undergoing the'mental reaction consequent on the
treatment of the sick, witnessing the distress of the bereaved,
all this when she feels herself about to become a mother, or
when her rest is disturbed by her child and she must endure
care and discomfort on its account." There is much more
to the same effect. Miss Crepaz does not remember
that the claims of society are found infinitely more
wearing both to mind and body than the regular
routine of a profession, however arduous. It has become
a truism that busy people have the most time. We think
experience will also show that it is the busy mothers who
see most of their children. Devotion to regular work never
need involve the withdrawal of time and attention from the
claims of family life; these claims and duties will always
tend to rank higher than ever as the standard of womanhood
is raised. It is the endless visiting for visiting's sake of
indifferent acquaintance, it is the struggle for recognition by
thoae a little richer or a little more important, it is the truly
miraculous absorption in the mysteries of the toilette, for
which there will be " no time " in the life of the busy woman.
She whose day is arranged on the lines of work which must!
be done, will, in ninety-nine cases out of a hundred find her
truest recreation in the ordering of her household and
her highest joys in those family ties which she is accused
of despising. That Miss Crepaz recognises to the full this
highest vocation of woman as a wife and mother is the main
merit of her little essay, which does not appear, however, to
be founded on a very wide experience of the subject
discussed.
Divers Dialogcbs. By U. and I.
(R. Hunter, Edinburgh.)
Ten dialogues by U. and I. are compressed into the small
compass of ninety-four pages. They may be described as
Email word pictures of a kind that will appeal to that large
portion of the community who like to be saved all mental
exertion and continuous mental effort. They are exclusively
society episodes, which speak for themselves, and are devoid
of all hampering descriptions of scenery and surroundings or
persons, in fact, they are arranged in dramatic fashion, name}
and scene of action being indicated above the dialogue. We
cannot imagine that this little book will find favour amongst
the earnest and thinking public, but the dialogues indicate a
certain amount of power, which we hope will be expended on
a worthier production by U. and I.

				

